# Bacterial Populations in Complementary Foods and Drinking-water in Households with Children Aged 10-15 Months in Zanzibar, Tanzania

**DOI:** 10.3329/jhpn.v27i1.3316

**Published:** 2009-02

**Authors:** Jacqueline K. Kung'u, Kathryn J. Boor, Shaali M. Ame, Nadra S. Ali, Anna E. Jackson, Rebecca J. Stoltzfus

**Affiliations:** ^1^ Division of Nutritional Sciences, Cornell University, Ithaca, NY 14853-6301, USA; ^2^ Department of Food Science, Cornell University, Ithaca, NY, USA; ^3^ Public Health Laboratory–Ivo de Carneri, Pemba Island, Zanzibar, Tanzania; ^4^ Weill Cornell Medical College, New York, NY, USA

**Keywords:** Bacteria, Coliform, Complementary foods, Enterobacteriaceae, Food safety, Water microbiology, Tanzania

## Abstract

Bacteria were quantified in samples of drinking-water and in two porridges prepared for infant-feeding [fortified instant soy-rice porridge (SRP) and cooked porridge (*Lishe bora*, LB)] in 54 households. Bacterial numbers were measured again after the porridges had been held at room temperature for four hours (T4). Findings were benchmarked against bacterial numbers in traditional complementary foods sampled from 120 households. Total bacteria, coliform, and Enterobacteriaceae counts were enumerated using Petrifilm™. The mean log bacterial numbers were the lowest for LB at T0 (2.24±0.84 cfu/g aerobic counts) and the highest for SRP at T4 (4.63±0.56 cfu/g aerobic counts). The total bacteria, coliform and Enterobacteriaceae counts were higher at T4 than at T0 for LB (p≤0.001); however, only the coliform and Enterobacteriaceae counts were higher at T4 than at T0 for SRP (p<0.001). Drinking-water, SRP0, traditional foods, and SRP4 all had the mean aerobic counts higher than the acceptable cut-off but the total bacterial count in SRP0 was not significantly (p=0.543) different from drinking-water. However, coliform and Enterobacteriaceae counts in SRP0 were higher than in drinking-water (p<0.001). Also, although the aerobic counts of SRP4 were not significantly (p>0.999) different from traditional foods, the coliform and Enterobacteriaceae counts were significantly higher in SRP4 than in traditional foods (p<0.001). It is, therefore, recommended that food safety concerns be addressed when improving complementary foods.

## INTRODUCTION

Beyond the age of six months, breastmilk alone is no longer sufficient to meet the nutritional demands of the growing infant, and other foods and liquids (complementary foods) should, therefore, be introduced (1,2). Empirical evidence, however, demonstrates that introduction of complementary foods in resource-poor settings can result in diets that are nutritionally inadequate and microbiologically unsafe, which can lead to multiple nutrient deficiencies (3-5) and the risk of exposure to foodborne pathogens and, consequently, to gastrointestinal illnesses (6-10).

Foodborne microbial agents can cause diarrhoeal diseases and ill-health in infants (9,11,12). Worldwide, diarrhoea is the second leading cause of death in children (after neonatal disorders) (13) and is a leading cause of growth-faltering and malnutrition (6). A great proportion (∼70%) of diarrhoeal episodes occur due to foodborne pathogens transmitted by unhygienic preparation of foods in households (6). Sociocultural constraints, such as social infrastructure, ignorance, incorrect beliefs and practices, taboos, poverty, insufficient food, lack of safe water and sanitation, and shortage of fuel and time may aggravate the situation (6). One possible strategy for overcoming inadequate resources, such as water and fuel, which are necessary to ensure adequate food handling, is the use of pre-prepared food mixtures (2).

In Zanzibar of the Tanzanian Republic and many other low-income countries, foods made from local grains predominate in the diet of children. At 10-15 months, food made from local tubers and green vegetables also may be incorporated, but to a small extent. Although breastfeeding may progress to 18 months, complementary foods are introduced as early as 0-3 month(s) in contrast to the recommended practice of exclusive breastfeeding for six months (14). This departure from the current recommendation is common in developing countries and has been attributed to cultural beliefs and ecological and socioeconomic constraints, including the work patterns of women (15,16). Consequently, malnutrition and disease rates in young children are high. For example, in Zanzibar, the site of this study, the prevalence of growth-faltering in this age-group is 46.2% for stunting, 9.5% for wasting, and 36% for underweight (14). These figures are higher than the sub-Saharan Africa averages of 41%, 7%, and 29% respectively (17). Malaria, diarrhoea, and pneumonia are the major causes of mortality among children aged less than five years in Zanzibar. Malaria due to *Plasmodium falciparum* is holoendemic with year-round transmission (18).

It is a great challenge, generally, to meet the micronutrient needs of children from feeding traditional local complementary foods in resource-poor settings. We, therefore, reasoned that introduction of alternative micronutrient-fortified complementary foods could reduce rates of stunting and micronutrient deficiencies in these areas. One such food is an instant soy-rice porridge (SRP), fortified with vitamin A, iron, and zinc, among other micronutrients.

During an initial acceptability trial of the SRP in Zanzibar, concern was raised regarding the microbiological quality of water used for reconstituting it. Although mothers were instructed to add cool, boiled water to the product to their desired consistency and feed without cooking, the field staff discovered that most mothers were not complying with the instruction for boiling water but were instead using water from taps, wells, or rainwater that was also used for drinking. Therefore, we measured bacterial populations to assess the hygienic condition of various infant-foods (19). Bacterial populations were measured in domestic drinking-water stored in household containers, SRP, and *Lishe bora* (LB), a commercial porridge available in this setting that is cooked prior to serving. Specifically, (a) bacterial populations were quantified in drinking-water, SRP, and LB given to infants in the households, (b) bacterial numbers were measured again in the same porridges at four hours post-preparation to provide an indication of the resulting bacterial quality of these foods if not consumed in one feeding, and (c) bacterial populations were measured in various complementary foods collected from the same households to enable benchmarking of the microbiological profiles of drinking-water, SRP, and LB within the context of the complementary foods typically fed to infants in Zanzibar.

## MATERIALS AND METHODS

### Study setting

The study was conducted on Pemba Island, Zanzibar, United Republic of Tanzania, which is located in the Indian Ocean, approximately 50 km off the Tanzanian coast. Pemba is mostly rural with only about 20-25% of its population (362,166 in 2002) living in urban areas. In addition to subsistence agriculture and fishing, the main economic activity is the clove industry, with more than 70% of Zanzibar's harvest being produced in Pemba. Most urban households have tap-water, although maintenance of utilities through routine quality/quantity analyses and chlorination does not occur. Rural households lack basic services, such as running tap-water and electricity. Household refrigerators are rare, even when electricity is available. Pemba Island has an equatorial climate with average monthly temperature ranging from 24 °C to 28 °C.

Permission to carry out the study was sought from local leaders (*Shehas*) in Zanzibar. Each mother gave verbal consent to provide water and food samples for the study.

### Study design

**Fig. 1.**
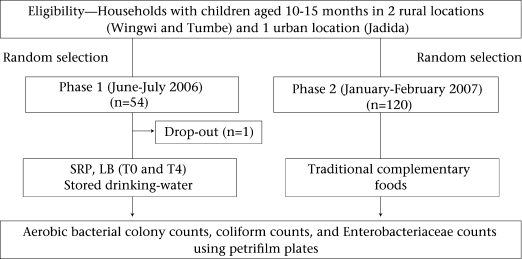
Study design

Households for sample collection were selected by sampling from a register of households with children aged 10-15 months in two rural locations—Wingwi and Tumbe—and one urban location—Jadida. These three study sites in the northern half of the island were purposively selected to maximize sample diversity. SRP, LB, and stored drinking-water were collected during phase 1 from a random sample of 54 households while samples of traditional complementary foods were collected in phase 2 from a different random sample of 120 households (Fig. 1).

LB=*Lishe bora*; SRP=Soy-rice porridge

### Sample collection and preparation

Phase 1 of sample collection involving SRP, LB, and water samples took place during June-July 2006 (Fig. 1). On each occasion, a field worker collected ∼10 mL of stored drinking-water and 10 g of SRP immediately after preparation and again at four hours after preparation, and LB immediately after preparation and again at four hours after preparation. Phase 2 collection of traditional complementary foods took place during January-February 2007. Foods were sampled at the time of feeding of the child. Samples were collected at different times of the day so as to capture the morning and mid-day feeding times. The item used for sample collection depended on the feeding mode used at the time of sample collection. For instance, if the mother used finger-feeding as the feeding mode, she was asked to use her hand to place the food sample in the sterile falcon tube used for sample collection. An effort was made to request the mother prior to collection not to prepare a special food for this exercise but to provide food that composed the regular diet of child. One complementary food sample was collected per household.

All samples were placed in sterile falcon tubes with tightly-fitting lids and kept on icepacks in a cooler box. This allowed immediate cooling of hot food samples from the households and reduced bacterial growth while samples were in transit. Samples were transported to the Public Health Laboratory—Ivo de Carneri where they were prepared and analyzed for bacterial populations within a day. When more than one type of food was given to a child simultaneously, the actual mixed portion as would be eaten by the child was collected. Additional information was obtained from the mother or caregiver on utensils used for feeding, time of food preparation, source of water for food preparation, whether the food was leftover, and if leftover food was reheated prior to feeding.

### Microbiological analyses

Food samples were plated for enumeration of aerobic colony counts, coliform counts, and Enterobacteriaceae counts (20). All the samples were evaluated using 3M™ Petrifilm™ aerobic count plates, coliform count plates, and Enterobacteriaceae count plates (3M Microbiology, St. Paul, MN, USA) according to the instructions of the manufacturer. Food samples were diluted in sterile distilled water (typically from 1:50 to 1:200) to enable colony enumeration. The diluted food sample was transferred to a sterile 3 M filtered homogenizer bag and homogenized for three minutes using a Stomacher machine (PBI International, Milan, Italy). The contents were allowed to settle, and then 1 mL of the liquid suspension was plated onto the appropriate Petrifilm™ according to the instructions of the manufacturer. The plates were incubated in stacks of no more than 20 at 35±1 °C for 48 hours for the aerobic count plates and for 24 hours for coliform and Enterobacteriaceae count plates.

At the Food Safety Laboratory of Cornell University, under sterile conditions, the SRP or LB flours were subjected to either no-heat treatment (assayed for total bacterial counts) or heat treatment (assayed for spore counts). Two samples of 25 g of each flour were added to 225 mL phosphate buffer and were mixed thoroughly for an initial 10^−1^ dilution. At time zero (T0), 10^−1^, 10^−2^ and 10^−3^ dilutions were prepared from one of the samples and spread-plated onto Brain Heart Infusion agar (BHI; Difco Laboratories, MD, USA). To assess the ability of bacteria present in the powdered sample to increase in number under room temperature conditions (i.e. simulating a situation in which the same batch of a product would be fed to a child over a number of hours), the original 10^−1^ dilutions were held on the bench at room temperature (approximately 25 °C) for four hours (T4) after which 10^−1^, 10^−2^, 10^−3^ and 10^−4^ dilutions were prepared and spread-plated onto BHI. Simultaneously, to measure numbers of spore-forming bacteria present in the flour, the second bottle of each product (the initial 10^−1^ dilution) was heat-treated at 80 °C for 12 minutes (T0), then 10^−1^, 10^−2^ and 10^−3^ dilutions were prepared and spread-plated onto BHI. The bottle that had been heat-treated was also held on the bench at room temperature for four hours (T4) after which 10^−1^, 10^−2^, 10^−3^ and 10^−4^ dilutions were prepared and plated onto BHI media. All the plates were incubated at 32 °C, and counts were obtained after 48 hours. Using the most probable number (MPN) technique, bacterial counts were determined.

Based on previously-reported criteria for assessing the bacterial quality of ready-to-eat foods, for the purposes of this study, upper limits for bacterial numbers were defined as: ≥10^4^ cfu/g for aerobic plate counts (20,21), ≥100 cfu/g for coliforms (21,22), and ≥100 cfu/g for Enterobacteriaceae (20).

### Analysis of data

All values were log_10_ transformed prior to statistical analyses. Geometric means are presented as mean log_10_ values±standard deviation. Analysis of variance (ANOVA) and Student's *t*-test statistics were used for comparing the geometric means of the bacterial counts. Pair-wise comparisons between each pair of means were examined using a post-hoc bonferroni test. Chi-square (χ^2^) tests were used for comparing proportions. Simple rank-ordering was done to benchmark bacterial numbers present in the water, SRP, and LB within the context of bacterial numbers present in the traditional complementary foods typically fed to children in Zanzibar. P values of <0.05 were considered statistically significant. All statistical analyses were carried out using the SPSS software (version 16).

## RESULTS

### Microbiology of water

Phase 1 of sample collection involved collection of samples from two rural locations (36 households) and one urban location (17 households). In general, tap-water was more accessible to urban households (30.2%) compared to rural locations (7.5%) (Table 1). Only about one-third (20/53) of the households used tap-water for the preparation of SRP and LB. In phase 2, more (67/120) households used tap-water than well-water (53/120) for the preparation of traditional foods in both rural and urban locations. Phase 2 of sample collection took place during the dry season; therefore, none of the households had access to rainwater (Table 1). Although there was a significant difference in the proportion of households that used different sources of water (p<0.001), there was no difference in bacterial counts by source of water used (rain vs tap vs well) for the preparation of complementary food (aerobic bacteria p=0.193, coliform p=0.861, and Enterobacteriaceae p=0.729, data not shown).

**Table 1.** Source of water used for preparation of complementary foods in phase 1 and 2

	Source of water∗							
Complementary food	Rain		Tap		Well		Total	
	No.	%	No.	%	No.	%	No.	%
Phase 1								
Soy-rice porridge								
Rural	3	5.7	4	7.5	29	54.7	36	67.9
Urban	0	0	16	30.2	1	1.9	17	32.1
Lishe bora								
Rural	3	5.7	4	7.5	29	54.7	36	67.9
Urban	0	0	16	30.2	1	1.9	17	32.1
Phase 2								
Traditional foods								
Rural	–		27	22.5	53	44.2	80	66.7
Urban	–		40	33.3	0	0	40	33.3

^∗Based on χ^2^ test, there is a statistical difference by source of water^

### Microbiology of SRP and LB from households in Zanzibar

The mean log bacteria counts for household drinking-water (n=53), LB (n=53), SRP (n=53), and traditional complementary foods (n=120) are shown in Table 2. It is notable that, in all counts, *Lishe bora* at T0 had the lowest bacterial counts while SRP at T4 had the highest counts. Also, in both foods, T4 counts were consistently significantly higher than corresponding T0 counts (p<0.05), except for SRP4 log aerobic counts which were not significantly different from SRP0 (p>0.999) (Table 2). There was no difference in bacterial counts by location (Tumbe vs Wingwi vs Jadida) from which the sample was collected [(aerobic bacteria p=0.933, coliform p=0. 952, and Enterobacteriaceae p=0.762), data not shown)].

**Table 2.** Mean log_10_ bacterial counts in water and complementary foods

Bacterial counts cfu/g†‡¶	Water and complementary food samples∗						p value
	Water (n=53)	LB0 (n=53)	LB4 (n=53)	SRP0 (n=53)	SRP4 (n=53)	Traditional foods (n=120)	
Aerobic bacteria	4.03±0.67^a^	2.24±0.84^b^	3.89±0.73^c^	4.36±0.48^a^	4.63±0.56^a^	4.58±1.02^a^	<0.001
Coliform	1.87±0.48^a^	1.71±0.08^a^	2.35±0.82^c^	2.80±0.87^b^	3.63±0.84^d^	2.40±1.02^c^	<0.001
Enterobacteriaceae	2.15±0.54^a^	1.73±0.12^a^	2.54±0.93^c^	3.10±0.85^b^	3.93±0.77^d^	2.58±1.17^c^	<0.001

∗LB0 is *Lishe bora* immediately after preparation; LB4 is *Lishe bora* 4 hours after preparation; SRP0 is soy-rice porridge immediately after preparation; and SRP4 is soy-rice porridge 4 hours after preparation

†Bacterial counts in mean Log_10_ cfu/g; the assay cannot detect values <50 cfu/g

‡Mean±SD, with ANOVA p value and post-hoc bonferroni test comparing water, LB0, and SRP0; LB0 and LB4; SRP0 and SRP4; LB4, SRP4, and traditional foods

¶Comparisons with unlike superscripts (a, b, c, and d) indicate significant differences (p<0.05) between subgroups; ANOVA=Analysis of variance; cfu=Colony-forming unit; LB=*Lishe bora*; SRP=Soy-rice porridge; SD=Standard deviation

### Are drinking-water, SRP, and LB better or worse than traditional complementary foods?

Simple rank-ordering of the mean bacterial counts of drinking-water, SRP, LB, and traditional complementary foods showed similar trends for aerobic bacteria, coliform and Enterobacteriaceae counts. To determine whether drinking-water, SRP, and LB are better or worse off than traditional foods, we present the results and post-hoc comparisons from the mean aerobic bacteria, coliform and Enterobacteriaceae counts. LB0 ranked 1^st^ (i.e. lowest) in the mean log aerobic bacteria (2.24±0.84 cfu/g), coliform (1.71±0.08 cfu/g) and Enterobacteriaceae (1.73±0.12 cfu/g) counts. After porridge was left in the household environment for four hours (LB4), LB4 ranked 2^nd^ in the mean log aerobic bacteria counts (3.89±0.73 cfu/g) with the mean counts significantly different (p<0.001) from LB0. It ranked 3^rd^ in the mean log coliform (2.35±0.82 cfu/g) and Enterobacteriaceae counts (2.54±0.93cfu/g), and these too were significantly different (p≤0.001) from LB0 (Table 2). Although drinking-water, SRP0, traditional foods, and SRP4 all had the mean aerobic bacteria counts higher than the acceptable cut-off, the total bacterial count in drinking-water was not significantly (p=0.543) different from SRP0. Also, the SRP4 aerobic bacteria counts were not significantly (p>0.999) different from traditional foods, but were significantly (p<0.001) different from LB4 (Table 2).

### Percentage of households with counts exceeding international guidelines on bacterial safety

The percentage of complementary food and water samples with aerobic bacteria, coliform, and Enterobacteriaceae exceeding the criteria for assessing the bacterial quality of ready-to-eat foods was the lowest for LB0, with 7.5% of samples exceeding limits for the aerobic bacteria plate counts, 1.9% exceeding limits for coliforms, and 3.8% exceeding limits for Enterobacteriaceae and was the highest for SRP4, with 92.5%, 92.5%, and 98.1% of the samples exceeding the respective limits (Table 3). In general, higher bacterial numbers and, therefore, larger proportions of samples exceeding bacterial limits were found in samples collected at T4 compared to those collected at T0. Although less than 10% of the LB0 exceeded these cut-offs, about half (49.1% aerobic bacteria contamination, 52.8% coliform contamination, 58.5% Enterobacteriaceae contamination) of these samples exceeded the cut-offs after a four-hour period in the households (LB4) (p<0.001) (Table 3). It is notable that there was a significant difference in the proportion of households exceeding these limits between water and SRP0 (aerobic bacteria p=0.008, coliform and Enterobacteriaceae p<0.001) (Table 3).

**Table 3.** Percentage of households with bacterial counts exceeding international guidelines

Bacterial counts cfu/g†‡¶	Water and complementary food samples∗						p value
	Water (n=53)	LB0 (n=53)	LB4 (n=53)	SRP0 (n=53)	SRP4 (n=53)	Traditional foods (n=120)	
Aerobic bacteria ≥104 cfu/g	50.9 (27)^a^	7.5 (4)^b^	49.1 (26)^d^	77.4 (41)^c^	92.5 (49)^e^	71.7 (86)^f^	<0.001
Coliform ≥100 cfu/g	35.8 (19)^a^	1.9 (1)^b^	52.8 (28)^d^	75.5 (40)^c^	92.5 (49)^e^	45.8 (55)^d^	<0.001
Enterobacteriaceae ≥100 cfu/g	50.9 (27)^a^	3.8 (2)^b^	58.5 (31)^d^	86.8 (46)^c^	98.1 (52)^e^	50.0 (60)^d^	<0.001

Figures in parentheses indicate numbers

∗LB0 is *Lishe bora* immediately after preparation; LB4 is *Lishe bora* 4 hours after preparation; SRP0 is soy-rice porridge immediately after preparation; and SRP4 is soy-rice porridge 4 hours after preparation

†Bacterial counts in mean Log_10_ cfu/g; the assay cannot detect values <50 cfu/g

‡% (n) with χ^2^-test p value comparing water, LB0, and SRP0; LB0 and LB4; SRP0 and SRP4; LB4, SRP4, and traditional foods

¶Comparisons with unlike superscripts (a, b, c, d, e, and f) indicate significant differences (p<0.05) between subgroups; cfu=Colony-forming unit; LB=*Lishe bora*; SRP=Soy-rice porridge

### Food safety laboratory analysis of bacterial contamination in SRP and LB flours

**Fig. 2.**
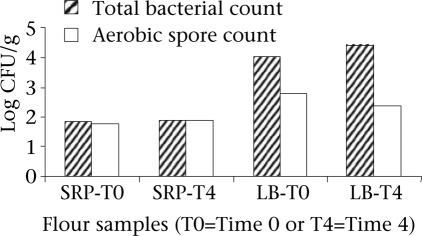
Bacterial quality of SRP and LB flours using MPN technique∗

The high bacterial counts in the infant-foods studied could be explained by the presence of bacterial spores or other bacteria in the SRP and LB flours. However, the total bacterial counts and aerobic spore counts were similar in the SRP flour at both T0 and T4, suggesting that the bacterial counts of the flours largely comprised spore-forming microbes. Dissimilar counts as in the LB flours suggest that the presence of other groups of bacteria, in addition to spore formers in the LB flours, contributed to the high bacterial counts in the LB flours (Fig. 2).

∗Viable organisms are enumerated using the MPN (most probable number) serial dilution technique; CFU=Colony-forming unit; *LB=Lishe bora*; SRP=Soy-rice porridge

### Description of traditional complementary foods

#### Type of sample

Due to a large variety of samples of complementary foods fed to children in Zanzibar Island, an effort was made to put these into major food groups to effectively describe them. The classification of major food groups consisted of starchy staples, purchased foods, protein sources, vegetable sources, and various combinations of these groups. The Appendix includes a detailed list of types of food samples that were classified into these major food groups, and Table 4 shows the bacterial counts by various characteristics. There was no statistical difference in bacterial counts between different types of food groups (aerobic bacteria p=0.441, coliform p=0.544, and Enterobacteriaceae p=0.500).

**Table 4.** Mean log_10_ bacterial counts in traditional complementary foods by various characteristics

Characteristics	Bacterial counts cfu/g				
	No.	%	Aerobic bacteria∗†	Coliform∗†	Enterobacteriaceae∗†
Types of sample‡					
Starchy staples	56	46.7	4.65±1.04	2.40±0.99	2.64±1.21
Purchased foods	28	23.3	4.28±0.96	2.34±0.90	2.48±1.03
Starchy staples + protein source	29	24.2	4.70±1.09	2.49±1.23	2.60±1.31
Starchy staples + protein source + vegetable	3	2.5	4.28±0.42	1.70±0.00	1.70±0.00
Starchy staples + vegetable	2	1.7	5.39±1.37	3.41±0.42	3.79±0.13
Other (protein source, fruit)	2	1.7	4.91±0.28	1.97±0.38	2.18±0.67
Storage time (hours)¶					
<4	89	74.2	4.48±0.99§	2.26±0.95∗∗	2.43±1.12∗∗
≥4	31	25.8	4.87±1.08	2.80±1.09	3.02±1.24
Leftover food¶					
Yes	17	14.2	5.29±1.05∗∗	3.03±1.16∗∗	3.20±1.30∗∗
No	103	85.8	4.47±0.98	2.30±0.96	2.48±1.13
Reheat and leftover‡¶					
Yes	2	11.8	3.72±0.23§	2.44±1.05	2.37±0.94
No	15	88.2	5.50±0.93	3.12±1.18	3.31±1.32
Feeding mode‡					
Finger-feeding	96	80.0	4.67±1.01	2.40±1.06	2.58±1.20
Spoon	13	10.8	4.24±0.90	2.35±0.70	2.56±1.08
Cup	7	5.8	4.54±0.85	2.40±0.77	2.67±1.03
Bottlefeeding	4	3.3	3.73±1.74	2.58±1.38	2.57±1.43
Source of water‡					
Tap	67	55.8	4.48±1.02	2.30±1.01	2.47±1.19
Well	53	44.2	4.71±1.02	2.54±1.01	2.73±1.15
Caregiver‡					
Mother	89	74.2	4.49±0.93	2.29±0.79	2.46±0.99
Sibling	10	8.3	4.79±1.01	2.36±1.30	2.62±1.36
Self	16	13.3	4.79±1.49	2.76±1.53	2.94±1.65
Other relatives	5	4.2	5.14±0.86	3.33±1.54	3.54±1.73

∗All values are mean+SD

†Bacterial counts in mean log_10_ cfu/g; the assay cannot detect values <50 cfu/g

‡Based on Student's *t*-test or ANOVA, there is no significant difference

¶Based on Student's *t*-test or ANOVA, there is significant difference

§Based on Student's *t*-test or ANOVA, p<0.1

∗∗Based on Student's *t*-test or ANOVA, p<0.05; ANOVA=Analysis of variance; cfu=Colony-forming unit; SD=Standard deviation

The staple foods of the Zanzibari diet are rice and cassava, which are usually eaten with small quantities of legumes, small fish, or green vegetables. More children (56/120) were fed starchy staples consisting of rice, porridge, cassava, *shelisheli* (breadfruit), potatoes, pancakes, sweet potatoes, pumpkins, plantains, or maize-flour *Ugali*. Meat and large fish are expensive and were, therefore, not regularly consumed. Fruits (mainly mangoes, pineapples, and oranges) tend to be seasonal. The primary weaning food was maize, although foods made from local tubers and fried dough (*maandazi*) were also introduced early (Table 4, Appendix).

**Appendix.** Grouping samples into food groups

Food group	Type of sample
Starchy staples	Rice, *uji* (porridge), cassava, *shelisheli* (breadfruit), potatoes, pancake, sweet potatoes, pumpkin, cassava with plantain, cassava with tea, breadfruit with tea, potatoes in coconut milk, potatoes with plantain, maize-flour *ugali* (stiff maize-meal) with potatoes in coconut milk, breadfruit in coconut milk, plantain, cassava in coconut milk, and maize-flour *kigali* (soft maize-meal)
Purchased foods	*Andazi* (fried wheat dough) with tea, bread with tea, *andazi* with *uji*, biscuit with tea, bread with milk, biscuit
Starchy staples + protein source	Rice with fish, rice with beans, potatoes with fish, rice with cowmilk, maize-flour *ugali* with fish, maize-flour *ugali* with beans, cassava with beans, plantain with *dagaa* (whitebait), breadfruit with whitebait, maize-flour *ugali*, beans with fish, cassava with fish, and potatoes with octopus
Starchy staples + protein source + vegetable	Rice, cassava green leaves, and fish; rice and *Mchicha* (spinach) with fish stew
Starchy staples + vegetable	Rice with sweet potato greens, and maize-flour *ugali* with *mchicha*
Other	Eggs and cooked papaya

#### Storage time

Although about half (66/120) of the samples were collected before noon, the time that had elapsed between food preparation and sample collection (storage time) varied greatly, ranging from 0 to 19.25 hour(s), with a mean storage time of 4.04±5.-05 hours. There was a statistical significant difference in coliform (p=0.011) and Enterobacteriaceae (p=0.016) counts but not in aerobic bacteria plate counts (p=0.065) in food with storage time of less than four hours versus those with storage time greater or equal to four hours (Table 4).

#### Leftover food

Seventeen of the 120 households reported that the food that we collected was leftover food (17/120). This was the food remaining from a meal eaten the day before sample collection. These samples consistently had higher means in aerobic bacteria (p=0.002), coliform (p=0.006) and Enterobacteriaceae (p=0.019) counts. Only two of the 17 households reported that they had reheated the leftover food before feeding it to the child. Reheated leftover food had lower mean counts than non-reheated food samples; this was, however, significantly (p=0.047) different only for aerobic bacteria counts.

#### Caregiver and mode of feeding

Among the households (n=120) sampled, the person feeding the child during sample collection was usually the mother (74.2%). Other caregivers reported were sibling (8.3%), children themselves (13.3%), or a relative (4.2%). Finger feeding was the most prevalent (80%) mode of feeding in these households, followed by use of utensils, such as spoon (10.8%), cup (5.8%), or bottle (3.3%). There was no difference in bacterial counts by caregiver (aerobic bacteria p=0.359, coliform p=0.064, and Enterobacteriaceae p=0.126) or by feeding mode (aerobic bacteria p=0.179, coliform p=0.985, and Enterobacteriaceae p=0.998) (Table 4).

## DISCUSSION

### Microbiology of water

Commercially-available fortified complementary foods are considered to be convenient and nutritious in developed countries, although their use has not been widespread because these are generally unaffordable by many households. Due to the high burden of malnutrition in children aged less than two years (23), there has been growing interest in increasing the availability and affordability of these products (24). Although SRP was developed to be within the means of many developing-country households and was considered by mothers to be convenient, addressing the dual risks of multiple micronutrient deficiencies and gastrointestinal illnesses presents a challenge. All sources of water examined in this study failed to meet the guidelines of the World Health Organization (WHO) for quality of drinking-water (25). Availability of safe water is central to a satisfactory practice of food hygiene. United Nations Children's Fund and WHO reported that strategies to reduce the contamination of complementary foods and prevent diarrhoea should focus on provision of safe water and safe preparation and storage of food (26,27). A number of authors reported that provision of safe water alone was not enough to control foodborne diseases, rather a multidisciplinary approach that addresses both environmental conditions (water supplies and sanitation) and promotes and protects breastfeeding and health education (hygiene promotion during food handling) is required (6,7,28).

### Microbiology of SRP and LB from households in Zanzibar and bacterial contamination in SRP and LB flours

It is acknowledged that contamination of complementary foods may occur as a result of poor hygiene of those who prepare food, household equipment, and the environment where the preparation of food takes place (29). High bacterial numbers in infant porridges held for four hours after preparation in the households suggest possible household contamination during this time period or the presence of spores or other bacteria capable of reproducing in hydrated flours. Our results suggest that bacteria other than spore-formers contribute to the high bacterial numbers found in the porridges. This conclusion is further supported by the coliform and Enterobacteriaceae numbers (which, by definition, are not spore-formers). Bacterial spores present in the SRP flours had similar counts at T0 and T4, implicating an external source of contamination, such as the environment or food handler as the explanation for the high counts in the household samples. Bacterial numbers should be very low in foods immediately after cooking, as we observed for LB soon after cooking (LB0). The presence of bacteria in LB0 indicates inadequate heat treatment or post-treatment contamination (30). Given the high bacterial counts in the water samples, it was not unexpected to see equally high counts in the instant SRP samples.

### Microbiology of traditional complementary foods

Bacterial counts of traditional complementary foods did not vary by food groups. This suggests that the level of contamination is typical for a particular household and not unique to the type of sample. In similar studies, contamination has been found to depend largely on food-hygiene practices and storage time (31,32). In addition, less variation has been found within than between households (33). Ready-to-eat foods obtained from street-vendors in Nigeria were reported to have two-fold higher bacterial numbers than home-prepared foods (34). Therefore, in the present study, we hypothesized that purchased foods might have a higher bacterial number than home-prepared foods. Purchased foods in our study included locally-prepared foods from market vendors and commercially-manufactured and packaged foods, such as biscuits and bread. However, bacterial numbers in purchased foods, which accounted for 23.3% of the collected samples, did not differ significantly from those in other food groups. Our data suggest that the household is a major source of food contamination.

### Storage time/leftover food

Our findings suggest that a four-hour time lapse between food preparation and food consumption can result in significantly-increased coliform and Enterobacteriaceae numbers in both LB and SRP and significant aerobic bacteria numbers in LB. Two studies in Bangladesh have reported increases in bacterial numbers in complementary foods at the household level with duration of storage (35,36). In particular, Henry *et al*. observed an increase in coliform counts when there was a more-than-four-hour delay between preparation and consumption of the weaning food (36). Although UNICEF recommends that food should be eaten without delay or thoroughly reheated (26), studies reported that leftover foods fed to infants were rarely reheated (37), and time and fuel were cited as the primary constraints preventing women from reheating these foods (22). We also found that a few Zanzibari mothers reheated leftover foods.

### Caregiver/feeding modes

Other risk factors for microbial contamination analyzed were the child's caregiver and the mode of feeding. Although the mean log bacterial counts were lower in samples collected from households where mothers are the persons who fed the child, there was no statistical difference in bacterial counts among the different caregivers or among the different feeding modes. In contrast, a Nigerian study found that mothers practised more favourable hygiene standards compared to other caregivers (31).

### Comparing traditional foods versus novel foods

LB0 had the lowest bacterial numbers as expected given that it is a cooked porridge. Since the households were asked to prepare it for the purposes of sample collection, it is also possible that they might have practised higher hygiene standards than usual, resulting in better ranking for LB0 than for similarly-cooked traditional complementary foods. The mean storage time for the traditional complementary food was 4.04±5.05 hours. SRP, a fortified instant porridge, was not expected to have better ranking than household drinking-water.

Bacterial numbers in the complementary foods reported here are high and are generally above the international guidelines for bacterial safety of complementary foods (21,38). However, our results are consistent with those of previous studies on the microbial quality of complementary foods, which also reported the presence of foodborne pathogens in infant formula (39-41) and in home-prepared complementary foods and water in other developing countries (31,33,37,42).

High bacterial counts in the complementary food samples reflect the poor microbiological quality in household drinking-water. In addition, household handling and storage increased risk due to bacterial reproduction during a time-lapse between food preparation and consumption and contamination from the household environment and the food-handler. Notwithstanding this risk, introduction of an instant complementary food would be advantageous in that it would save time and fuel in its preparation. Its ease of preparation might increase the feeding frequency and, therefore, increase intake. We conclude that hygiene and sanitation interventions should be integrated with the introduction of SRP, or other instant porridges. Further, appropriate and sustainable household food-processing, preparation and storage techniques must be developed and adapted to local conditions to reduce the microbial contamination of foods.

## ACKNOWLEDGEMENTS

The authors thank the Public Health Laboratory–Ivo de Carneri for facilitating the study and contributing to field work and laboratory analyses. They also thank InstaLIFE (InstaLIFE International, Weiser, ID and InstaPRO International, Des Moines, IA) for providing the soy-rice porridge used in the study and Mario Einaudi Center and the Global Health Program at Cornell University and First Presbyterian Church of Ithaca for providing funding for international travel and to carry out the study.

## References

[1] Pan American Health Organization (2001). Guiding principles for complementary feeding of the breastfed child.

[2] World Health Organization (1998). Complementary feeding of young children in developing countries: a review of current scientific knowledge.

[3] Kimmons JE, Dewey KG, Haque E, Chakraborty J, Osendarp SJ, Brown KH (2005). Low nutrient intakes among infants in rural Bangladesh are attributable to low intake and micronutrient density of complementary foods. J Nutr.

[4] Dewey KG, Brown KH (2003). Update on technical issues concerning complementary feeding of young children in developing countries and implications for intervention programs. Food Nutr Bull.

[5] Hotz C, Gibson RS (2001). Complementary feeding practices and dietary intakes from complementary foods amongst weanlings in rural Malawi. Eur J Clin Nutr.

[6] Motarjemi Y, Kaferstein F, Moy G, Quevedo F (1993). Contaminated weaning food: a major risk factor for diarrhoea and associated malnutrition. Facts Infant Feed.

[7] Sheth M, Dwivedi R (2006). Complementary foods associated diarrhea. Indian J Pediatr.

[8] Lanata CF (2003). Studies of food hygiene and diarrhoeal disease. Int J Environ Health Res.

[9] Brown KH, Black RE, Lopez de Romana G, Creed de Kanashiro H (1989). Infant-feeding practices and their relationship with diarrheal and other diseases in Huascar (Lima), Peru. Pediatrics.

[10] Mosha TCE, Laswai HS, Tetens I (2000). Nutritional composition and micronutrient status of home made and commercial weaning foods consumed in Tanzania. Plant Foods Hum Nutr (Dordrecht, Netherlands).

[11] Motarjemi Y (2000). Research priorities on safety of complementary feeding. Pediatrics.

[12] World Health Organization (1989). Research on improving infant feeding practices to prevent diarrhoea or reduce its severity: memorandum from a JHU/WHO meeting. Facts Infant Feed.

[13] Black RE, Morris SS, Bryce J (2003). Where and why are 10 million children dying every year?. Lancet.

[14] United Nations Children's Fund (2001). Situation analysis for women and children in Zanzibar 2001.

[15] Sellen DW (2001). Weaning, complementary feeding, and maternal decision making in a rural east African pastoral population. J Hum Lact.

[16] Sellen DW (1998). Infant and young child feeding practices among African pastoralists: the Datoga of Tanzania. J Biosoc Sci.

[17] United Nations Children's Fund (2006). The state of the world's children 2006.

[18] Mebrahtu T, Stoltzfus RJ, Chwaya HM, Jape JK, Savioli L, Montresor A (2004). Low-dose daily iron supplementation for 12 months does not increase the prevalence of malarial infection or density of parasites in young Zanzibari children. J Nutr.

[19] Motarjemi Y, Kaferstein F, Moy G, Quevedo F (1993). Contaminated weaning food: a major risk factor for diarrhoea and associated malnutrition. Bull World Health Organ.

[20] Gilbert RJ, de Louvois J, Donovan T, Little C, Nye K, Ribeiro CD (2000). Guidelines for the microbiological quality of some ready-to-eat foods sampled at the point of sale. Commun Dis Public Health.

[21] Codex Alimentarius: code of hygienic practice for powdered formulae for infants and young children. FAO/WHO Food Standards, 2008. http://www.codexalimentarius.net/web/more_info.jsp?id_sta=11026.

[22] Kimmons JE, Brown KH, Lartey A, Collison E, Mensah PP, Dewey KG (1999). The effects of fermentation and/or vacuum flask storage on the presence of coliforms in complementary foods prepared for Ghanaian children. Int J Food Sci Nutr.

[23] Black RE, Morris SS, Bryce J (2003). Where and why are 10 million children dying every year?. Lancet.

[24] Fiedler J, Saunders M, Sanghvi T (2007). What are the costs of interventions? Program costs vary by type of intervention, country setting, and methodology. Food Nutr Bull.

[25] World Health Organization (1997). Guidelines for drinking-water quality.

[26] United Nations Children's Fund (1989). Facts for life—hygiene.

[27] World Health Organization (1996). Basic principles for the preparation of safe food for infants and young children.

[28] Kaferstein F (2003). Foodborne diseases in developing countries: aetiology, epidemiology and strategies for prevention. Int J Environ Health Res.

[29] Sheth M, Patel J, Sharma S, Seshadri S (2000). Hazard analysis and critical control points of weaning foods. Indian J Pediatr.

[30] World Health Organization (2004). Guidelines for drinking-water quality.

[31] Iroegbu CU, Ene-Obong HN, Uwaegbute AC, Amazigo UV (2000). Bacteriological quality of weaning food and drinking water given to children of market women in Nigeria: implications for control of diarrhoea. J Health Popul Nutr.

[32] Afifi ZE, Nasser SS, Shalaby S, Atlam SA (1998). Contamination of weaning foods: organisms, channels, and sequelae. J Trop Pediatr.

[33] Imong SM, Rungruengthanakit K, Ruangyuttikarn C, Wongsawasdii L, Jackson DA, Drewett RF (1989). The bacterial content of infant weaning foods and water in rural northern Thailand. J Trop Pediatr.

[34] Ehiri JE, Azubuike MC, Ubbaonu CN, Anyanwu EC, Ibe KM, Ogbonna MO (2001). Critical control points of complementary food preparation and handling in eastern Nigeria. Facts Infant Feed.

[35] Black RE, Brown KH, Becker S, Alim AR, Merson MH (1982). Contamination of weaning foods and transmission of enterotoxigenic *Escherichia coli* diarrhoea in children in rural Bangladesh. Trans R Soc Trop Med Hyg.

[36] Henry FJ, Patwary Y, Huttly SR, Aziz KM (1990). Bacterial contamination of weaning foods and drinking water in rural Bangladesh. Epidemiol Infect.

[37] Potgieter N, Obi CL, Bessong PO, Igumbor EO, Samie A, Nengobela R (2005). Bacterial contamination of *Vhuswa*—a local weaning food and stored drinking-water in impoverished households in the Venda region of South Africa. J Health Popul Nutr.

[38] Commission Regulation (EC) No. 2073/2005 of 15 November 2005 on microbiological criteria for foodstuffs. Official Journal of the European Union. http://eur-lex.europa.eu/LexUriServ/site/en/oj/2005/l_338/l_33820051222en00010026.pdf.

[39] Estuningsih S, Kress C, Hassan AA, Akineden O, Schneider E, Usleber E (2006). Enterobacteriaceae in dehydrated powdered infant formula manufactured in Indonesia and Malaysia. J Food Protec.

[40] Forsythe SJ (2005). Enterobacter sakazakii and other bacteria in powdered infant milk formula. Maternal Child Nutr.

[41] Becker H, Schaller G, von Wiese W, Terplan G (1994). Bacillus cereus in infant foods and dried milk products. Int J Food Microbiol.

[42] Morais TB, Sigulem DM, de Sousa Maranhao H, de Morais MB (2005). Bacterial contamination and nutrient content of home-prepared milk feeding bottles of infants attending a public outpatient clinic. J Trop Pediatr.

